# A critical role of solute carrier 22a14 in sperm motility and male fertility in mice

**DOI:** 10.1038/srep36468

**Published:** 2016-11-04

**Authors:** Shin-ya Maruyama, Momoe Ito, Yuusuke Ikami, Yu Okitsu, Chizuru Ito, Kiyotaka Toshimori, Wataru Fujii, Keiichiro Yogo

**Affiliations:** 1 Laboratory of Animal Physiology, Faculty of Agriculture, Shizuoka University, Shizuoka, Japan; 2 Department of Reproductive Biology and Medicine, Graduate School of Medicine, Chiba University, Chiba, Japan; 3 Department of Animal Resource Sciences, Graduate School of Agricultural and Life Sciences, The University of Tokyo, Tokyo, Japan; 4 Laboratory of Animal Physiology, Academic Institute, College of Agriculture, Shizuoka University, Shizuoka, Japan

## Abstract

We previously identified solute carrier 22a14 (Slc22a14) as a spermatogenesis-associated transmembrane protein in mice. Although Slc22a14 is a member of the organic anion/cation transporter family, its expression profile and physiological role have not been elucidated. Here, we show that Slc22a14 is crucial for sperm motility and male fertility in mice. Slc22a14 is expressed specifically in male germ cells, and mice lacking the *Slc22a14* gene show severe male infertility. Although the overall differentiation of sperm was normal, *Slc22a14*^−/−^ cauda epididymal spermatozoa showed reduced motility with abnormal flagellar bending. Further, the ability to migrate into the female reproductive tract and fertilise the oocyte were also impaired in *Slc22a14*^−/−^ spermatozoa. The abnormal flagellar bending was thought to be partly caused by osmotic cell swelling since osmotic challenge or membrane permeabilisation treatment alleviated the tail abnormality. In addition, we found structural abnormalities in *Slc22a14*^−/−^ sperm cells: the annulus, a ring-like structure at the mid-piece–principal piece junction, was disorganised, and expression and localisation of septin 4, an annulus component protein that is essential for the annulus formation, was also impaired. Taken together, our results demonstrated that Slc22a14 plays a pivotal role in normal flagellar structure, motility and fertility in mouse spermatozoa.

Solute carrier (SLC) transporters are a large protein family involved in the transport of various substances, such as ions, amino acids, sugars, metabolites and xenobiotics (e.g., drugs). The SLC family consists of over 350 genes classified into 52 subfamilies by functional and sequential homology^1^,^2^. Unlike ATP-binding cassette (ABC) transporters, which directly require ATP, SLCs transport substrates by facilitated diffusion or secondary active transport using ion gradients generated by Na, K-ATPase. SLC transporters play important roles in diverse physiological functions, including nutrient uptake in the intestine, glucose reabsorption in the kidney, excretion of xenobiotics in the liver and regulation of neurotransmission in the brain. In addition, defects in SLC functioning are closely associated with human diseases^2^,^3^ such as glucose/galactose malabsorption^4^, familial renal glucosuria^5^ and amyotrophic lateral sclerosis^6^. To date, mutations in 20% of SLC transporters have been associated with Mendelian disease, and it is predicted that disease-related mutations will be found in more SLC transporter genes^2^. Owing to their biological and pharmacological importance and the advantage of their being located on the cell surface, SLC transporters are promising as targets for drug discovery and/or as biomarkers^2^.

More than 10% of couples experience infertility, and approximately half of the cause is attributed to men^7^; however, the genetic cause of male infertility remains to be elucidated. To understand the molecular basis for sperm differentiation and function and to find potential targets for fertility drugs, we previously screened for spermatogenesis-associated transmembrane protein genes in mice^8^ and identified 53 genes, including *Slc22a14*, a member of the Slc22 transporter subfamily. The Slc22 family consists of 23 genes that act as organic anion/cation transporters. Each member selectively transports organic anions or organic cations where they usually show broad substrate specificity, at least *in vitro*, with varying affinity. Experimental evidence of their physiological function has been accumulated for some members of the Slc22 family. For example, Slc22a1 (OCT1) is localised at the basolateral membrane of epithelial cells in the liver, kidney and intestine in rats and mediates excretion of many cationic drugs and xenobiotics^9^,^10^,^11^. Although *Slc22a1*-deficient mice are viable, fertile and healthy, liver uptake and direct intestinal excretion of substrate organic cations is impaired^12^. In addition, SLC22A12 (URAT1) has been identified as a human urate transporter localised in the luminal membrane of the renal proximal tubules^13^, and it has been revealed that *SLC22A12* mutations are associated with renal hypouricaemia^13^,^14^. In contrast, only limited information has been provided regarding Slc22a14 so far.

*SLC22A14* was first identified as *ORCTL4* (organic cation transporter-like 4) with a high similarity to organic cation transporters in humans^15^. *SLC22A14* is predicted to encode an approximately 70-kDa protein with 12 transmembrane regions and is well conserved in many mammals. Northern blotting analysis showed that human *SLC22A14* expression seemed ubiquitous but is most prominent in the testis^15^. More interestingly, *Slc22a14* is a candidate gene responsible for male infertility in oligotriche mutant mice. Oligotriche is a recessive mutation that affects the coat and male fertility in mice, and the homozygous mutant (*olt/olt*) shows alopecia in the inguinal region and male infertility owing to defective spermatogenesis^16^. Runkel *et al*.^17^ have reported that the oligotriche mutation is a deletion of 234 kb on distal chromosome 9 and the locus contains 6 genes, including *Slc22a14*. They also found that *Slc22a14* is expressed in the wild-type testis but not in the *olt/olt* mutant testis. These data suggest a possible role of Slc22a14 in male fertility; however, the expression profile of Slc22a14 in the testis has not been characterised and the physiological function of Slc22a14 remains to be elucidated. In this report, we investigated the expression, cellular localisation and physiological role of Slc22a14 in male fertility in mice.

## Results

### Slc22a14 is specifically expressed in male germ cells and localised in the principal piece of the tail

**Figure 1:**
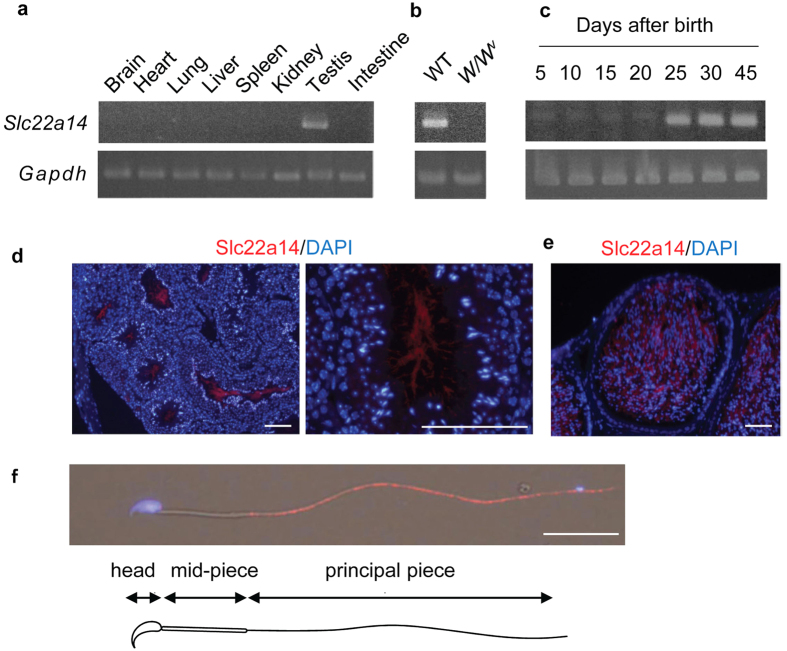
Expression of *Slc22a14* in mouse tissues. (**a**) Expression analysis of *Slc22a14* mRNA in various mouse tissues using RT-PCR. The *Slc22a14* signal was detected only in testis. (**b**) Expression of *Slc22a14* mRNA in wild-type (WT) and *W/W *^v^ mouse testis, which lacks germ cells. (**c**) Expression of *Slc22a14* mRNA during postnatal testicular development. (**d**) Testicular localisation of Slc22a14. Sections of mouse testis were immunohistochemically stained using anti-Slc22a14 antibody (red). Nuclei were stained with 4′,6-diamidino-2-phenylindole (DAPI, blue). *Left panel*: lower magnification; *right panel*: higher magnification. Bar, 50 μm. (**e**) Immunohistochemical staining of cauda epididymis using anti-Slc22a14 antibody. Bar, 25 μm. (**f**) Intracellular localisation of Slc22a14 in mouse spermatozoa. Spermatozoa from cauda epididymis was stained as in (**d**). Slc22a14 is predominantly localised to the principal piece. Bar, 20 μm. The regions of head, mid-piece, and principal piece are shown.

The expression of *Slc22a14* mRNA in various mouse tissues was analysed using reverse transcription-polymerase chain reaction (RT-PCR), and its expression was found to be specific in testis (Fig. 1a). *Slc22a14* expression was not observed in *W/W *^v^ mouse testis, which lacks germ cells, suggesting that *Slc22a14* is expressed only in germ cells (Fig. 1b). In addition, we examined the change of expression of *Slc22a14* during the first wave of spermatogenesis and found that it began to be expressed in testes at 25 days of age (Fig. 1c). Since the first spermatids appear around day 20 in the first wave of spermatogenesis^18^, these results indicate that *Slc22a14* expression begins in developing spermatids. Next, we investigated the localisation of Slc22a14 in mouse testes using immunofluorescence staining. The Slc22a14 signals were mainly observed in the seminiferous tubule lumen, suggesting that Slc22a14 is localised in the sperm flagella (Fig. 1d). Supporting this, the Slc22a14 signals were also detected in the cauda epididymal lumen (Fig. 1e). The specificity of the antibody was confirmed using *Slc22a14*^−/−^ mouse testis (Supplementary Fig. S1). To more precisely determine the intracellular localisation, epididymal spermatozoa were stained with anti-Slc22a14 antibody. The immunofluorescence signals were predominantly detected in the principal piece of tail, but not in the head (Fig. 1f).

### Deficiency of *Slc22a14* gene leads to severe infertility

**Figure 2:**
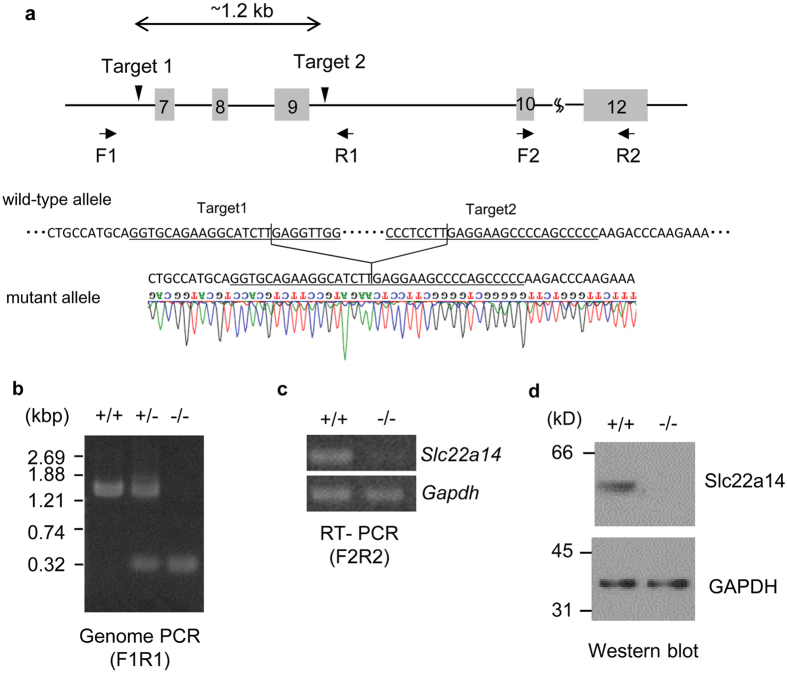
Generation of *Slc22a14*-deficient mice using the CRISPR/Cas9 system. (**a**) Schematic representation of targeting strategy. *Upper panel*: partial structure of *Slc22a14* gene on chromosome 9. The grey box indicates the location of the exon. The location of two targeted sites (targets 1 and 2) and two primer pairs (F1R1 and F2R2) used for PCR analysis are also shown. *Lower panel*: nucleotide sequence of wild-type allele in the vicinity of two target sites (underlined) and sequence analysis of the mutant allele of *Slc22a14* heterozygous mice. The desired deletion of the *Slc22a14* gene was confirmed. (**b**) Genome PCR analysis in wild-type (+/+), heterozygous (+/−), and homozygous (−/−) mutant mice using primers F1 and R1. (**c**) Expression analysis of *Slc22a14* mRNA using RT-PCR in wild-type or homozygous mutant testis. (**d**) Western blotting of testis extract using anti-Slc22a14 antibody.

To investigate the physiological role of Slc22a14, we generated a *Slc22a14* gene-deficient mouse using the CRISPR/Cas9 system. Ablation of the gene at the desired region was confirmed by genomic DNA sequencing (Fig. 2a) and genome PCR (Fig. 2b). In addition, non-specific genome modifications were not observed in at least two potential off-target sequences (Supplementary Fig. S2). Expression of *Slc22a14* mRNA and its product were prominently decreased in homozygous mutant mice (Fig. 2c,d). *Slc22a14*-deficient mice were born approximately at the expected ratio according to Mendel’s law (46 +/+, 105 +/− and 35 −/−) and no apparent abnormalities were observed in growth or behaviour, including alopecia; however, *Slc22a14*^−/−^ male mice showed severe infertility (Table 1) despite that a vaginal plug was usually observed. All female mice that mated with wild-type male mice delivered, and the mean litter size was 8.5. In contrast, among 10 female mice that mated with *Slc22a14*^−/−^ male mice, only one mice delivered. On the other hand, female fertility was not affected as shown in *olt/olt* mice.

**Figure 3:**
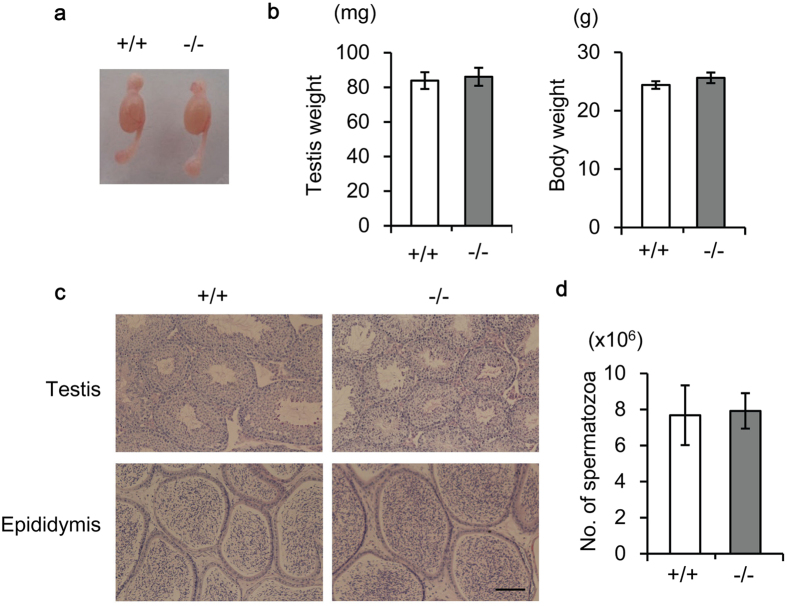
Morphological and histological analysis of *Slc22a14*-deficient testis and epididymis. (**a**) Representative images of testis and epididymis of wild-type and *Slc22a14*^−/−^ mice. (**b**) Testis weight (*left*) and body weight (*right*) of 13-week-old wild-type and *Slc22a14*^−/−^ mice. Data are presented as average ± SE (n = 5). (**c**) Hematoxylin and eosin staining of sections of testis and cauda epididymis. Bar, 100 μm. (**d**) Number of cauda epididymal spermatozoa in wild-type and *Slc22a14*^−/−^ mice. Data are presented as average ± SE (n = 4).

To investigate the cause of male infertility, we performed morphological and histochemical analyses of testis and epididymis. There was no significant difference in gross appearance or weight of testes between wild-type and homozygous mutant mice (Fig. 3a,b). In addition, all stages of germ cells, from spermatogonia to spermatozoa, were observed in seminiferous tubules, and the seminiferous epithelial cycle also appeared to be normal in *Slc22a14*^−/−^ mice (Fig. 3c). Further, cauda epididymides were filled with mature spermatozoa in *Slc22a14*^−/−^ mice (Fig. 3c), and the number of epididymal sperm was not different from that of wild-type mice (Fig. 3d). These results suggest that spermatogenesis is essentially normal in *Slc22a14*^−/−^ mice.

### *Slc22a14* ^−/−^ sperm show impaired motility and abnormal flagellar bending

**Figure 4:**
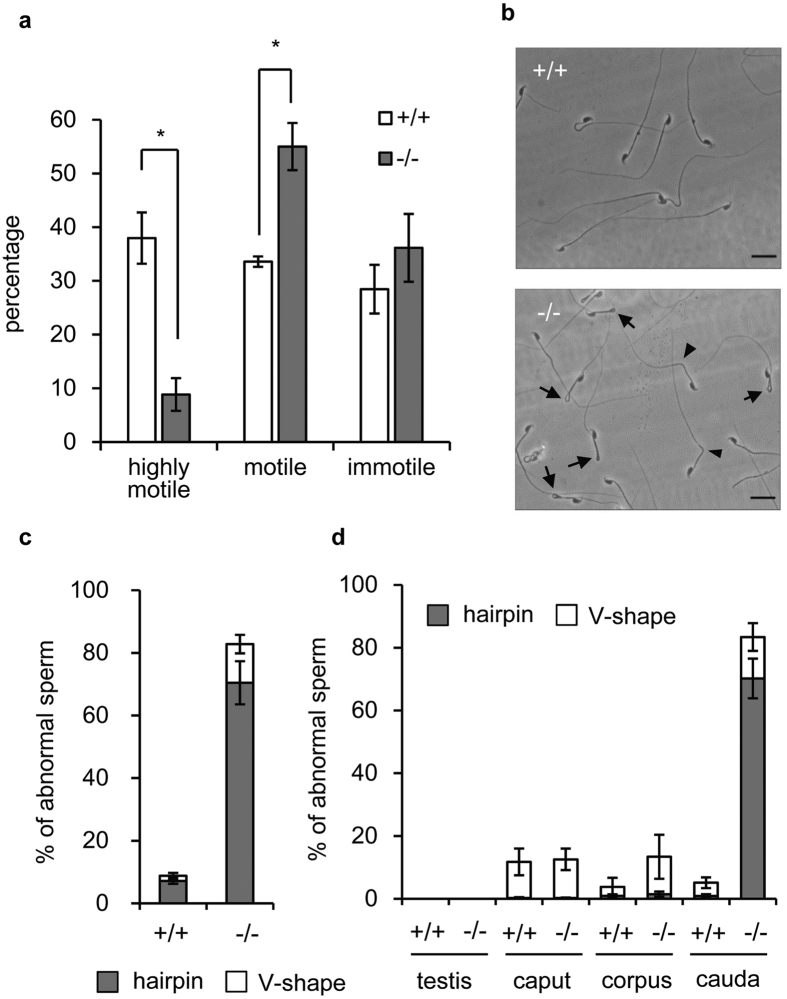
Impaired motility and abnormal flagellar angulation in *Slc22a14*^−/−^ spermatozoa. (**a**) Sperm motility analysis in wild-type and *Slc22a14*^−/−^ mice. Videos of sperm were captured and the motility of each sperm was categorised. More than 200 sperm were counted in each experiment. Data are presented as average ± SE (n = 3). Asterisks indicate significant differences between wild-type and homozygous mutants (*p* < 0.01). The *p*-values were calculated using the unpaired *t*-test from the mean values of data using Microsoft Excel. (**b**) Morphology of wild-type and *Slc22a14*^−/−^ spermatozoa. Two types of abnormal flagellar angulation (hairpin-type [arrow] and V-shape type [arrowhead]) were observed in *Slc22a14*^−/−^ spermatozoa. Bar, 20 μm. (**c**) Percentage of cauda epididymal sperm with abnormal tails. More than 100 spermatozoa were counted in each experiment. Data are presented as average ± SE (n = 3). (**d**) Analysis of sperm morphology *in situ*. Spermatozoa were released from fixed testis or epididymis and the number of sperm with abnormal tails were counted under microscopy. Data are presented as average ± SE (n = 3).

Next, we investigated the motility of epididymal sperm. Movies were captured and motility of sperm were categorised into three groups: highly motile with progressive motility, motile but with weak or no progressive motility, and immotile. As shown in Supplementary Movies S1 and S2 and Fig. 4a, there were significantly fewer highly motile sperm in *Slc22a14*^−/−^ mice. During this observation, we noticed that most *Slc22a14*^−/−^ sperm showed abnormal flagellar bending (Fig. 4b,c). The *Slc22a14*^−/−^ sperm tails were mostly folded at 180° (hairpin-shaped), but some were folded to a lesser degrees (V-shape). The flexed point seemed to represent a boundary between the mid-piece and principal piece (Fig. 4b). These results suggest that reduced motility was caused by the flagellar abnormality. Next, we investigated the region where flagellar angulation occurs *in situ*. As shown in Fig. 4d, although the flagella of the testicular sperm or caput and corpus epididymal sperm were almost straight, many of the cauda epididymal sperm showed abnormal bending. This result suggests that the specific microenvironment of cauda epididymis or accumulation of physical stress during epididymal transit caused the abnormal flagellar bending in *Slc22a14*^−/−^ mice.

### *Slc22a14*-deficient sperm show impaired migration into the uterus and fertilisation

**Figure 5:**
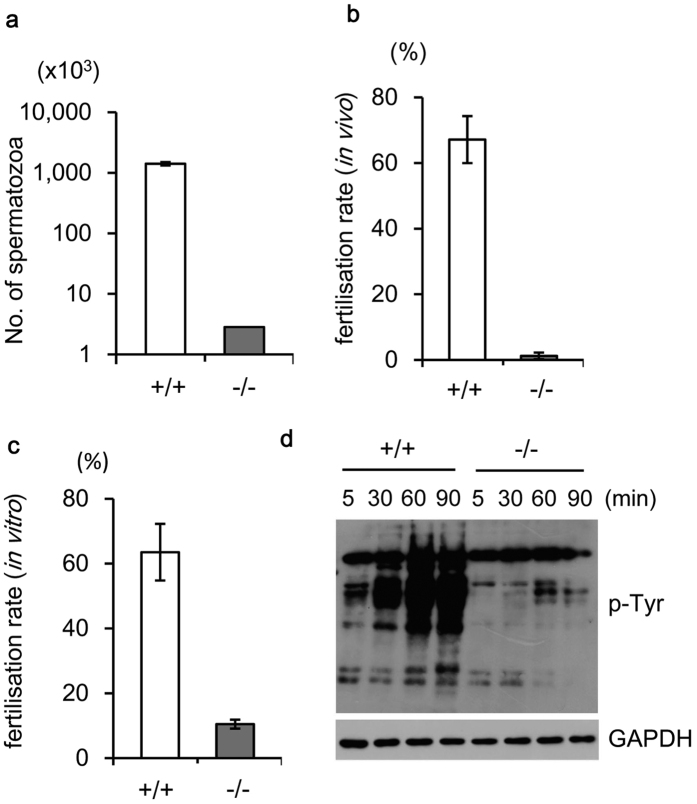
Fertilising ability of *Slc22a14*-deficient sperm. Wild-type or *Slc22a14*^−/−^ male mice were mated with wild-type female mice, and the number of spermatozoa in the uterus horns 2.5 h after of coitus (**a**) or the number of two-cell embryos recovered from oviducts 1.5 days after coitus (**b**) was counted. Data indicate the average of three experiments. Error bars represent standard errors. (**c**) Fertilisation rate *in vitro* using wild-type or *Slc22a14*^−/−^ spermatozoa and wild-type oocytes. The number of two-cell embryos were counted 24 h after insemination. Data are presented as average ± SE (n = 3). (**d**) Capacitation status of sperm. Sperm were incubated in TYH medium for the indicated times, and protein tyrosine phosphorylation (p-Tyr) levels were monitored using western-blotting.

We further investigated where the problem occurs during the fertilisation process in *Slc22a14*^−/−^ mice. First, the ability to migrate into the female reproductive tract was examined. As shown in Fig. 5a, the number of sperm recovered from uterus after copulation was extremely low in *Slc22a14*^−/−^ mice (<1% of that of wild-type mice). Consistent with this, the percentage of embryos fertilised *in vivo* was also lower in *Slc22a14*^−/−^ mice (Fig. 5b). The fertilisation rate of *Slc22a14*^−/−^ sperm was also apparently lower even with *in vitro* fertilisation (Fig. 5c), suggesting that *Slc22a14*^−/−^ sperm cannot fertilise the oocyte efficiently even if they could reach the fertilisation site. Finally, we investigated capacitation of *Slc22a14*^−/−^ sperm. Capacitation is the functional change of sperm necessary for fertilisation and can be detected as an increase in intracellular protein tyrosine phosphorylation^19^. As a result, when sperm were incubated in capacitating conditions, the intracellular protein tyrosine phosphorylation level was increased in wild-type sperm, but no increase was apparent in *Slc22a14*^−/−^ sperm (Fig. 5d), indicating that neither motility nor capacitation is normal in *Slc22a14*^−/−^ sperm and that these synergistic effects cause severe infertility in *Slc22a14*-deficient mice.

### Abnormal flagellar bending is partly caused by osmotic cell swelling

**Figure 6:**
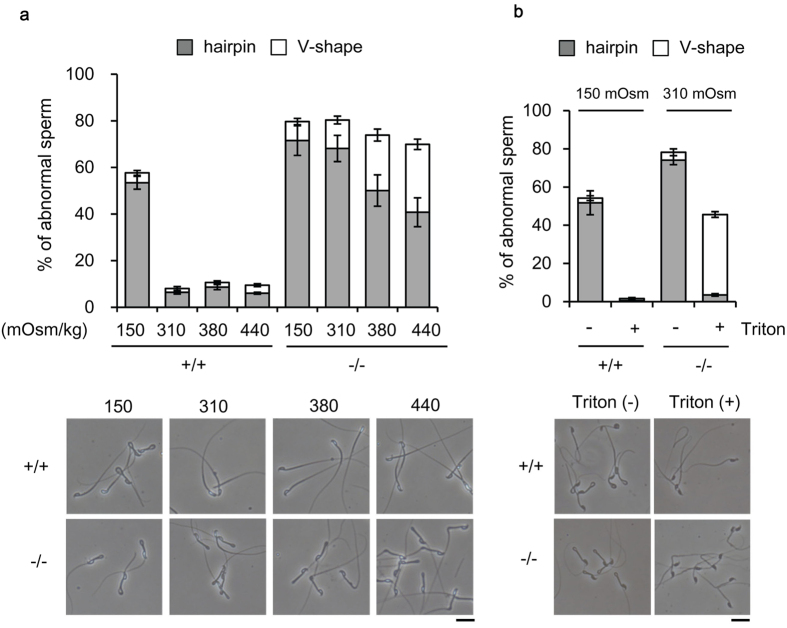
Effect of osmolality on abnormal flagellar bending. (**a**) Wild-type and *Slc22a14*^−/−^ cauda epididymal spermatozoa were incubated for 30 min in media with varying osmolality and the percentages of spermatozoa with abnormal tails were quantified (*upper panel*). Representative photographs of sperm after incubation in each condition are shown in the *lower panel*. (**b**) Effect of membrane permeabilisation on flagellar angulation. Wild-type and *Slc22a14*^−/−^ spermatozoa were incubated in media with the indicated osmolality for 30 min followed by a treatment with or without 1% Triton X-100 for 1 min. Sperm with abnormal tails were counted (*upper panel*). Representative photographs of sperm with or without permeabilisation are shown in the *lower panel*. All data are presented as average ± SE (n = 3). Bar, 20 μm.

Hairpin-like abnormal flagellar angulation is often observed along with osmotic cell swelling^20^. When wild-type sperm were incubated in 150 mOsm/kg (corresponding to half-osmolality of plasma) medium, abnormal hairpin bending was induced, whereas there was no apparent change when the medium osmolality was increased (Fig. 6a). Since Slc22a14 is a solute carrier transporter, we hypothesised that the lack of Slc22a14 would affect the osmolality of sperm and examined the effect of osmolality on the tail shape of *Slc22a14*^−/−^ sperm. The angulation of the tail was alleviated as the osmolality of the medium was increased (Fig. 6a). Namely, hairpin-shape sperm were decreased and V-shape sperm were increased in hyperosmotic conditions. A similar result was obtained after membrane permeabilisation treatment using Triton X-100 (Fig. 6b), suggesting that osmotic cell swelling is a cause of the abnormal flagellar bending in *Slc22a14*^−/−^ sperm. However, although membrane permeabilisation completely restored the hypotonic-induced hairpin bending to the straight form in wild-type sperm, the restoration in *Slc22a14*^−/−^ sperm was partial (Fig. 6b), indicating that another irreversible structural problem also existed in *Slc22a14*^−/−^ sperm tail.

### The annulus is disorganised in *Slc22a14* ^−/−^ sperm

**Figure 7:**
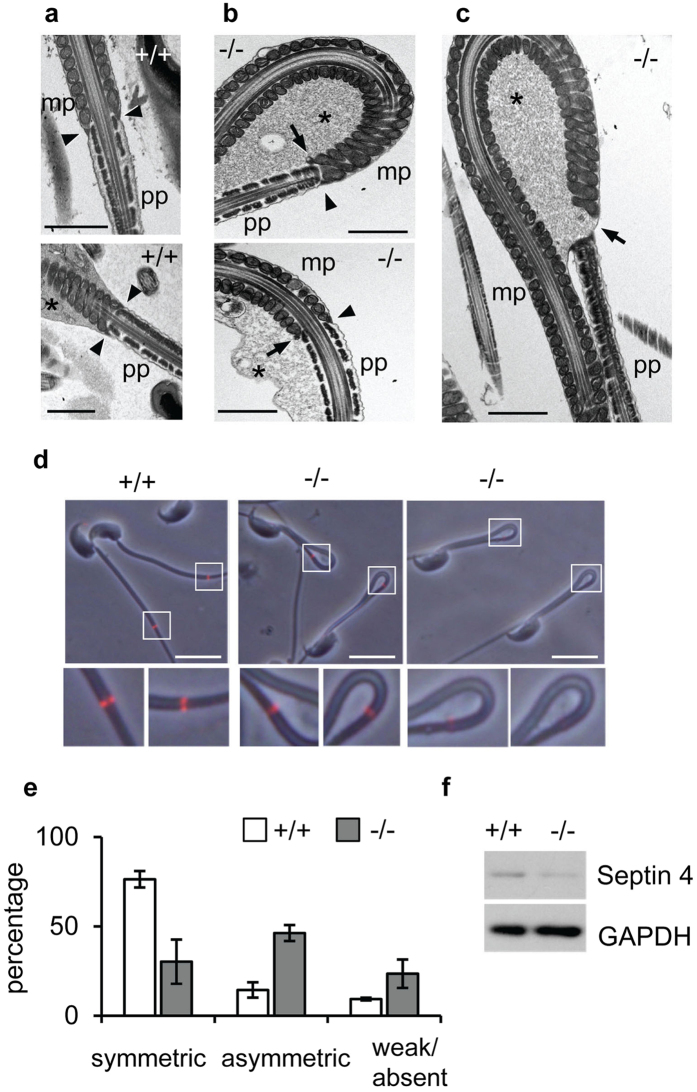
Abnormality of the annulus structure in *Slc22a14*^−/−^ spermatozoa. Electron microscopic analysis of cross-sections of wild-type (**a**) or *Slc22a14*^−/−^ (**b**,**c**) spermatozoa. The arrowhead, arrow and asterisk indicate normal annulus, detached annulus, and cytoplasmic droplets, respectively. Mp, mid-piece; pp, principal piece. Bar, 1 μm. (**d**) Immunohistochemical staining of septin 4 in wild-type or *Slc22a14*-deficient sperm. The two dots of septin 4 staining are frequently asymmetric (*middle*) or weak or absent (*right*) in *Slc22a14*^−/−^ sperm. Bar, 20 μm. (**e**) Quantification of localisation pattern of septin 4 shown in (**d)**. Data are presented as average ± SE (n = 3). (**f**) Expression analysis of septin 4 in wild-type and *Slc22a14*^−/−^ spermatozoa using western blotting.

We investigated the ultrastructure of flagellum using electron microscopy and found that the annulus is disorganised in *Slc22a14*^−/−^ sperm. The annulus is a ring-like structure composed of electron-dense filamentous materials that is localised at the mid-piece–principal piece boundary and closely associated with the plasma membrane (Fig. 7a). In *Slc22a14*^−/−^ sperm, the annulus is often detached from the membrane (Fig. 7b, arrow), and occasionally, structural breakage at the mid-piece–principal piece junction was observed (Fig. 7c). In addition, presumably owing to this annulus disorganisation, retention of cytoplasmic droplets was also observed in many *Slc22a14*^−/−^ sperm (Fig. 7b,c). It has been shown that septin 4 is an annulus component protein which is essential for annulus formation^21^,^22^. We therefore investigated the localisation of septin 4 in *Slc22a14*^−/−^ sperm. As shown in Fig. 7d,e, the immunofluorescence signals of septin 4 are usually observed as symmetrical pairs of dots in wild-type sperm. In contrast, in *Slc22a14*^−/−^ sperm, a significant portion of the signal was not symmetrical and sometimes weak or absent. Consistent with this, expression of septin 4 was slightly decreased in *Slc22a14*^−/−^ sperm (Fig. 7f). Since it has been shown that the annulus/septin 4 plays an important role in flagellar integrity and sperm motility^21^,^22^, these results suggest that disorganisation of the annulus is also a cause of the morphological and functional abnormality of *Slc22a14*^−/−^ sperm.

## Discussion

Slc22a14 was found to be expressed in a testis-specific manner and its gene products localised to the principal piece of spermatozoa. We investigated the physiological role of Slc22a14 using knockout mice and found that *Slc22a14*-deficient male mice showed severe infertility. Although the differentiation process in testis appeared to be normal, cauda epididymal spermatozoa in *Slc22a14*^−/−^ mice showed impaired motility with abnormal tails. Accordingly, despite normal mating behaviour, few sperm could enter the uterus, and the ability to fertilise was apparently lower in *Slc22a14*^−/−^ sperm. These results demonstrate that Slc22a14 is crucial for sperm motility and male fertility. To our knowledge, this is the first report showing the physiological function of Slc22a14.

One of the striking features of *Slc22a14*^−/−^ sperm is an abnormal flagellar bending. As shown in Fig. 6a, this characteristic abnormal bending could be induced in normal sperm when exposed to hypotonic conditions. Besides the hypotonic stress, inhibition or deletion of ion channels has been shown to induce the hairpin-like bending. For example, Na, K-ATPase α4-null sperm^23^ or SLO3-null sperm^24^,^25^ show the characteristic hairpin-shape abnormality with impaired motility and male fertility. This abnormal tail bending is thought to be attributed to the cell swelling caused by water influx. Supporting this, ablation of aquaporin3, a putative water efflux channel, results in the same abnormality^26^. Since Slc22a14 is a solute carrier, it is plausible that its deficiency affects osmolality in sperm and causes water influx. In fact, a hyperosmotic challenge and membrane permeabilisation treatment alleviated the abnormality (Fig. 6a,b), suggesting that osmotic cell swelling is a cause of tail abnormality in *Slc22a14*^−/−^ sperm.

In addition, we found that the annulus is disorganised in *Slc22a14*^−/−^ sperm. The annulus is a ring-like structure composed of several septins, members of a GTP-binding protein family. Among them, septin 4 is essential for annulus formation, mechanical integrity of flagella, and motility in sperm; septin 4-deficient sperms completely lack the annulus and have abnormal tails with sharp bending at the mid-piece–principal piece boundary^21^,^22^. In addition to the structural abnormalities, septin 4-deficient sperms have functional defects such as lower ATP consumption^22^ and decreased capacitating ability^21^, which reduce sperm motility and fertility. Owing to these sperm abnormalities, septin 4-null male mice are completely infertile. In this study, we found that the annulus was frequently disorganised in *Slc22a14*^−/−^ spermatozoa. In addition, we demonstrated that expression and localisation of septin 4 was also impaired. These results suggest that impairment of the annulus/septin 4 is also involved in the structural and functional abnormalities in *Slc22a14*^−/−^ spermatozoa. On the other hand, since expression of septin 4 was not completely lost in *Slc22a14*^−/−^ sperm (Fig. 7f), it is not clear whether only the decreased levels of septin 4 is responsible for the abnormalities. It is possible that other annulus component proteins also reduce in expression in *Slc22a14*^−/−^ sperms.

The mechanism by which the deletion of *Slc22a14* leads to abnormity of the annulus/septin 4 remains to be elucidated. One possibility is that prolonged physical stress caused the annulus disorganisation. Since abnormal flagellar angulation occurs at the cauda epididymis (Fig. 4d) and spermatozoa can be stored there beyond 30 days^27^, the annulus of *Slc22a14*^−/−^ sperm would be continuing to receive the physical stress such as membrane tension made by osmotic cell swelling and tail bending for a long period, which thereby may lead to the annulus disorganisation or destruction. We could not exclude the possibility that deletion of *Slc22a14* directly affects formation of the annulus or expression of septin 4; thus, further investigation is necessary to clarify the mechanism of decreased expression of septin 4 and/or disorganisation of the annulus in *Slc22a14*^−/−^ spermatozoa.

As described above, we found that abnormal hairpin bending arose in the cauda epididymis *in situ*. This indicates that the osmotic pressure of sperm is relatively higher than that of the luminal fluid of the cauda epididymis in *Slc22a14*^−/−^ mice. Since Slc22a14 is expressed specifically in sperm but not in cauda epididymal epithelial cells (Fig. 1), the imbalance of osmotic pressure may be caused by elevated osmolality in sperm. Although the molecular nature of the solute transport of Slc22a14 is unknown, Slc22a13, the most similar member of the Slc22 family to Slc22a14, is known to function as a unidirectional efflux transporter of aspartate, taurine, and glutamate^28^, and thus, Slc22a14 may have an analogous function in substrate transport. In this regard, glutamate is notable as a possible substrate for Slc22a14. The concentration of glutamate in epididymal fluid is high in the caput and corpus epididymis (50.23 and 19.48 mM, respectively) but low in the cauda epididymis (0.47 mM) in rats^29^. On the other hand, the intracellular glutamate concentration ranges from 1 to 10 mM in many cell types^30^,^31^. Therefore, it is presumed that the concentration gradient acts as force for glutamate efflux in cauda epididymal spermatozoa. If Slc22a14 is involved in a unidirectional efflux of glutamate, the osmolality of sperm lacking Slc22a14 could be increased in cauda epididymis and osmotic cell swelling could be induced. We are currently investigating this possibility.

In addition to the flagellar abnormality, *Slc22a14*^−/−^ sperm showed impaired capacitation. Capacitation is triggered by an influx of bicarbonate ions^32^ which directly activate soluble adenylate cyclase^33^. The intracellular cAMP concentration is then increased, protein kinase A is activated, and a series of reactions including activation of protein tyrosine kinase occurs downstream of protein kinase A^19^,^34^. A possible explanation for impaired capacitation in *Slc22a14*^−/−^ sperm is a change of membrane potential. Slc22a14 belongs to the organic anion/cation transporter family and some members of the SLC family act as symporters/antiporters of sodium ions. Thus, the lack of Slc22a14 could affect membrane potential and thereby inhibit the transport of bicarbonate ions. Otherwise, disorganisation of the annulus structure could affect localisation/function of bicarbonate transporters. In sperm, several bicarbonate transporters are expressed with specific intracellular localisation, and in flagellum, two bicarbonate transporters, Slc26a3 and Slc26a6, are expressed and localised to the mid-piece but not to the principal piece^35^. Since the annulus also functions as a membrane diffusion barrier that compartmentalises membrane proteins to specific cellular domains^36^,^37^, disorganisation of the annulus may affect the localisation and function of those transporters. Similarly, the localisation and function of Slc26a8 could be disturbed in *Slc22a14*^−/−^ sperm. Slc26a8 is a testis-specific anion transporter which is co-localised to the annulus. Since Slc26a8 is essential for capacitation^38^, impaired capacitation in *Slc22a14*^−/−^ sperm may reflect dysfunction of Slc26a8. Regarding this, intriguingly, the phenotype of *Slc22a14*^−/−^ mice is well concordant with that of *Slc26a8*^−/−^ mice. Namely, *Slc26a8*-deficient sperm shows reduced motility and abnormal flagellar angulation with a disorganised annulus, whereas the overall sperm differentiation process is normal^38^. Thus, a functional interaction may exist between Slc22a14 and Slc26a8. Aside from such a possibility, however, further studies are necessary to elucidate the mechanism through which loss of Slc22a14 impairs capacitation.

As described in the *Introduction*, Slc22a14 is a candidate gene responsible for male infertility in oligotriche (*olt/olt*) mutant mice. However, the reproductive phenotype apparently differed between *olt/olt* and *Slc22a14*^−/−^ mice. Namely, spermatogenesis is impaired and mature spermatozoa are almost absent in the epididymis of *olt/olt* mice, whereas spermatogenesis is essentially normal and mature spermatozoa are observed in *Slc22a14*-deficient mice. Therefore, another gene must be responsible for male infertility in oligotriche mice. The deleted locus in the oligotriche mutant is located at 234 kb on distal chromosome 9 and contains six genes including *Ctdspl, Vill, Plcd1, Dlec1, Acaa1b* and *Slc22a14*. The *Plcd1*^39^ and *Acaab1*^40^ genes were shown to be dispensable for male fertility using knockout mice, leaving three genes, *Ctdspl, Vill* and *Dlec1*, as potential candidates. We are currently investigating to identify which is the causative gene in *olt/olt* mice.

The reduced motility and flagellar abnormalities are distinctive features observed in idiopathic asthenozoospermia and teratozoospermia in humans. These disorders are major causes of male infertility, but the genetic cause is not fully understood. Since *Slc22a14* is conserved in humans and expressed predominantly in the testis, *SLC22A14* may be involved in the pathogenesis of such diseases. In this regard, many single-nucleotide polymorphisms in the human *SLC22A14* gene are found in the NCBI database, and in fact, a splice polymorphism which may cause the production of a truncated protein lacking a large part of SLC22A14 has been found in a Japanese population^41^. Whether this polymorphism is associated with male fertility is unknown, but future work should investigate the relationship between *SLC22A14* polymorphism and the pathogenesis of asthenozoospermia or teratozoospermia in humans.

## Methods

### Animals

Laboratory animals were purchased from Japan SLC Co. Ltd (Hamamatsu, Japan). All animal experiments were approved by the Institutional Committee for Experimental Animal Care and Use of Shizuoka University and the University of Tokyo and carried out in accordance with the Act on Welfare and Management of Animals and the Guidelines for Proper Conduct for Animal Experimentation (Science Council of Japan).

### Production of *Slc22a14* knockout mice

*Slc22a14*-deficient mice were generated using the CRISPR/Cas9 system as reported previously^42^ with slight modifications. For convenient genotyping, two target sequences were designed to eliminate exon 7 to exon 9: GGTGCAGAAGGCATCTTGAGGTTGG and GGGGCTGGGGGCTTCCTCAAGGAGGG (Proto-spacer adjacent motif sequences are underlined). DNA fragments that contain the target sequence with a T3 promotor and long guide RNAs were synthesised by PCR and inserted into a pGEM-T Easy vector. These guide RNA vectors and the CAS9 vector (Addgene ID: 48625) were linearised and transcribed *in vitro* using RNA polymerase. The RNA transcripts were purified by ethanol precipitation and suspended in ribonuclease-free water. RNA solutions were microinjected into the cytoplasm of fertilised eggs of C57BL/6N mice in the pronuclear stage, and the eggs were transferred into the oviduct of pseudopregnant ICR mice. After birth, the genotype was determined by genomic PCR. Considering the possibility of mosaicism in the F0 mouse, and to fix the mutation, one heterozygous F1 mouse was used as the founder.

### RT-PCR and genomic PCR

Isolation of total RNA from various mouse tissues and the reverse transcription reaction was carried out using ISOGEN (Nippon gene) and ReverTra Ace (TOYOBO) as previously described^43^. For genotyping analysis, genomic DNA was isolated from the tail tip using standard phenol/chloroform extraction. PCR was carried out with KOD FX Neo DNA polymerase (TOYOBO) according to the manufacturer’s instructions. PCR products were separated by agarose gel electrophoresis and the signals were visualised by ethidium bromide staining. The primers used for the analysis are listed in Supplementary Table S1.

### Antibodies

Rabbit polyclonal anti-Slc22a14 antibody was raised against a keyhole limpet hemocyanin-conjugated peptide corresponding to the C-terminal region of mouse Slc22a14 (^615^PKMDLPVQSLKAQPP^629^). Peptide synthesis, animal immunisation, and collection of sera were performed by Sigma-Aldrich Japan. The antibody was purified by affinity chromatography using an antigen peptide-conjugated column. Anti-GAPDH monoclonal antibody, anti-human septin 4 (N) antibody, and anti-phosphorylated tyrosine antibody (4G10) were purchased from WAKO Pure Chemical Industries, Immuno-Biological Laboratories (IBL), and Merck Millipore, respectively. Horseradish peroxidase-conjugated and Alexa Fluor-conjugated secondary antibodies were purchased from Santa Cruz Biotechnology and Life Technologies, respectively.

### Sodium dodecyl sulphate- polyacrylamide gel electrophoresis and western blotting

Mouse tissues were isolated and homogenised in buffer containing 50 mM Tris-Cl (pH 7.4), 150 mM NaCl, 2 mM EDTA, 2 mM phenylmethylsulfonyl fluoride, 2 mM Na_3_VO_4_, 20 mM NaF and 1% Triton X-100. The lysates were cleared by centrifugation at 20 000 *g* for 20 min at 4 °C, and protein concentrations were measured using the BCA protein assay kit (Pierce). The lysates were mixed with equal volumes of 2× sample buffer and incubated at 37 °C for 30 min (Slc22a14) or at 100 °C for 5 min (others). To prepare the sperm extract, cauda epididymal sperm were released into modified Krebs-Ringer bicarbonate solution (TYH medium) and incubated at 37 °C for the indicated times. Sperm were then collected in tubes and washed twice with phosphate-buffered saline (PBS) by centrifugation at 5 000 × *g* for 5 min. Finally, the pellet was resuspended in sample buffer and incubated at 100 °C for 5 min. The proteins were loaded on polyacrylamide gels and transferred onto polyvinylidene difluoride membranes. Western blotting was performed using standard procedures. The signals were visualised by chemiluminescence using homemade enhanced chemiluminescence solution containing 4-iodophenylboronic acid^44^.

### Histochemical analysis

Mouse tissues were fixed in Bouin’s solution and embedded in paraffin, and 4-μm-thick sections were cut from the paraffin block and mounted on poly-l-lysine–coated glass slides. The sections were then deparaffinised, rehydrated, and stained with hematoxylin and eosin. For immunohistochemical staining, the sections were heated for 5 min in citric acid buffer (pH 6.0) using a pressure cooker for the antigen retrieval. These sections were then blocked for 1 h in 5% skim milk/PBS, followed by incubation with primary antibody for 1.5 h. After washing with PBS, the samples were incubated with Alexa Fluor 488- or Alexa Fluor 546-conjugated secondary antibodies (Life Technologies) for 1 h. Nuclei were stained with 4′,6-diamidino-2-phenylindole (DAPI). The signals were detected by epifluorescence microscopy (Olympus IX-70 or BX-60) equipped with a digital camera. For immunohistochemical analysis of sperm, epididymal spermatozoa were released into the TYH medium, washed with PBS, fixed with 3.8% paraformaldehyde, and attached onto poly-l-lysine–coated glass slides. For staining Slc22a14, epididymal spermatozoa in TYH medium were directly smeared onto glass slides and then fixed with ice-cold methanol for 2 min. The spermatozoa were permeabilised with 0.2% Triton X-100/PBS and blocked with 5% skim milk/PBS for 1 h. Immunohistochemical staining was then similarly performed.

### Evaluation of male fertility

One 10-week-old male mouse was caged with two 8-week-old B6D2F1 wild-type female mice for 2 weeks. Female mice were checked daily for a vaginal plug and separated if pregnant. Litter sizes were recorded on delivery.

### Video capture of sperm and motility analysis

Epididymal spermatozoa from mature mice were incubated in TYH medium at 37 °C for 0.5 h, and cell suspensions were placed in a pre-warmed (37 °C) 10-μm-high chamber (Kitazato Corporation). Video was captured by a digital camera attached to a camera port of an Olympus IX-70 microscope. The motility of sperm was manually categorised into three groups (highly motile with progressive motility, motile but with week or no progressive motility and immotile) by monitoring the videos. More than 100 sperm were checked in each experimental group.

### *In vitro* fertilisation

Female mice were intraperitoneally injected with pregnant mare serum gonadotropin and 48 h later with human chorionic gonadotropin (hCG). Fourteen hours after the hCG injection, cumulus–oocyte complexes were collected from the oviduct ampulla and transferred into 200-μl drops of TYH medium covered with mineral oil. Spermatozoa were isolated from the cauda epididymides and incubated in TYH medium for 1 h. The capacitated spermatozoa were then incubated with cumulus–oocyte complexes at a concentration of 2 × 10^5^/ml for 6 h. After washing, eggs were cultured in M16 medium supplemented with 4 mg/ml bovine serum albumin for 24 h and the number of two-cell embryos was counted.

### Assessment of fertilisation *in vivo* and sperm migration into the uterus

Female mice were superovulated as described above and mated with male mice after the hCG injection. The oviducts were removed 1.5 days after mating (plug day = 0.5) and cut using a 26-gauge needle in PBS. Released eggs were collected under the stereomicroscope and the number of two-cell embryos counted. To analyse the migration of ejaculated sperm into the uterus, mice were checked every 30 min after mating and killed 2 hours after vaginal plug formation. The uteruses were removed and flushed three times with TYH medium containing 0.5% Triton X-100 using a 26-gauge needle. The number of sperm were then counted using a hematocytometer.

### Morphological analysis of sperm *in situ*, osmotic challenge, and permeabilisation treatment of sperm

Dissected testes and epididymides are fixed in 10% formaldehyde in PBS at 4 °C for 24 h. After washing with PBS medium, the tissues were minced with scissors, the resulting cell suspensions were mounted and the number of sperm with abnormal flagella was counted. In osmotic challenge assays, cauda epididymal spermatozoa were incubated in TYH media with varying osmolality adjusted by NaCl for 30 min. The spermatozoa were then collected by centrifugation and fixed with 3.8% paraformaldehyde for 15 min. After washing with PBS, spermatozoa were mounted on glass slides and monitored by microscopy. To examine the effect of permeabilisation on flagellar bending, epididymal spermatozoa were incubated in TYH medium for 30 min and then incubated in 1% Trion X-100 containing TYH medium for 1 min. The sperm samples were then similarly prepared.

### Electron microscopic analysis of sperm

Electron microscopic analysis was performed essentially as previously described^45^. Cauda epididymal spermatozoa were released into TYH medium and dispersed for 30 min. The spermatozoa were collected in tubes and centrifuged at 5000 × *g* for 5 min. After the supernatant was discarded, spermatozoa were fixed with 2.5% glutaraldehyde/phosphate buffer (pH 7.4). The spermatozoa were then post-fixed with 1% osmium tetroxide and embedded in Epon resin 812. Ultrathin sections were made and stained with uranyl acetate and lead citrate. The sections were observed using a JEOL JEM-1200 EX transmission electron microscope (JEOL).

## Additional Information

**How to cite this article**: Maruyama, S.-y. *et al*. A critical role of solute carrier 22a14 in sperm motility and male fertility in mice. *Sci. Rep.* **6**, 36468; doi: 10.1038/srep36468 (2016).

**Publisher’s note:** Springer Nature remains neutral with regard to jurisdictional claims in published maps and institutional affiliations.

## Supplementary Material

Supplementary Information

Supplementary Movie-S1

Supplementary Movie-S2

## Figures and Tables

**Table 1:** Male fertility of *Slc22a14*-deficent mice.

**Genotype**	**No. of females mated**	**No. of litters**	**Mean litter size**	**No. of births**
*Slc22a14*^+/+^	8	8	8.5	68
*Slc22a14*^−/−^	10	1	1	1

Male mice of the indicated genotype were mated with wild-type BDF1 female mice for 2 weeks, and number of litters, number of births and mean litter size were counted. In each experiment, one male mouse was mated with two female mice (n = 4 wild-type; n = 5 homozygous mutants).
