# Supplementary material for: A critical role of solute carrier 22a14 in sperm motility and male fertility in mice
Source: Sci Rep. 2016 Nov 4;6:36468. doi: 10.1038/srep36468 (PMC5095606; doi:10.1038/srep36468)
Supplement: Supplementary Information [file srep36468-s1.pdf]

Supplementary information

A critical role of solute carrier 22a14 in sperm motility and male fertility in mice

Shin-ya Maruyama, Momoe Ito, Yuusuke Ikami, Yu Okitsu, Chizuru Ito, Kiyotaka Toshimori, Wataru Fujii, and Keiichiro Yogo

Supplementary Fig. S1

Slc22a14/DAPI

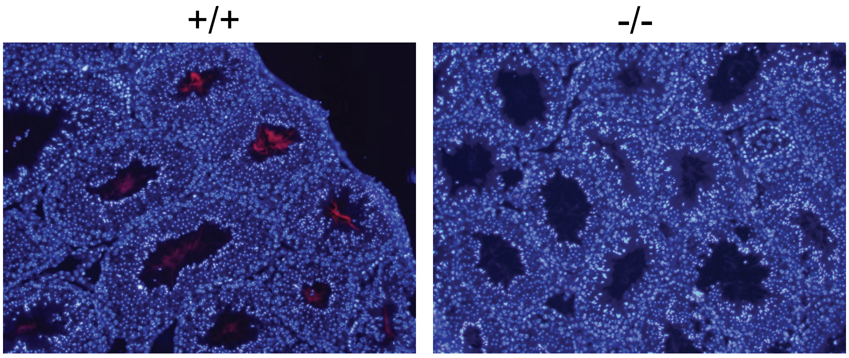

Figure S1. Specificity of anti-Slc22a14 antibody.
Immunohistochemical staining of wild-type (+/+) and *Slc22a14*-deficient (-/-) mouse testis using anti-Slc22a14 antibody (red). Nuclei were stained with 4',6-diamidino-2-phenylindole, dilactate (DAPI, blue).

Supplementary Fig. S2

Chromosome 3:
36270485-36270509

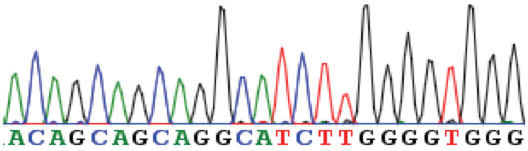

Slc22a14 target 1 <u>GGTGCAGAAGGCATCTTGAGGTTGG</u>

Chromosome 1:
87314659-87314683

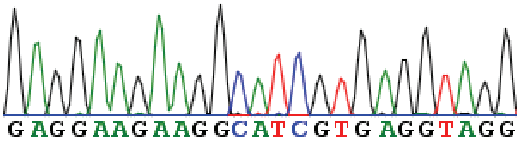

Slc22a14 target 1 <u>GGTGCAGAAGGCATCTTGAGGTTGG</u>

Chromosome 15:
79827961-79827986

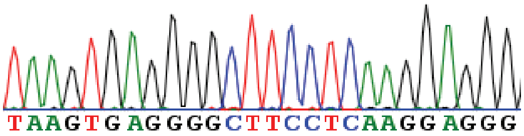

slc22a14 target 2 <u>GGGGCTGGGGGCTTCCTCAAGGAGGG</u>

Chromosome 18:
40387086-40387111

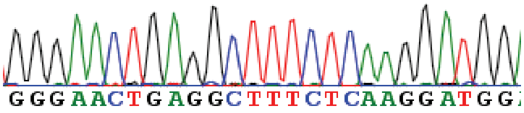

slc22a14 target 2 <u>GGGGCTGGGGGCTTCCTCAAGGAGGG</u>

Figure S2. Sequence analysis of potential off target sites.
Potential off-target sites for each target sequence (underlined) were searched with TagScan (http://ccg.vital-it.ch/tagger/tagscan.html). The two sites with the highest similarity were selected, the region was amplified by PCR and the sequences were analysed by direct sequence analysis. The chromosome number and region of the sites are indicated above the waveform data. There were no deletions or insertions in all sites.

Supplementary Table S1

Table S1. Primer sequences used in this study

Gene	Primer pair	Primer sequence
Slc22a14	F1	5′-GCTTAGAGATGATTAGAGCAGGTAAAGAGAC-3′
(genotyping)	R1	5′-CCACTAAAGACCATTACTGCTACCATCTAC-3′
Slc22a14	F2	5′-ATCCTGACGCTCATGCTTGC-3′
(RT-PCR)	R2	5′-CAAGGTCATGTTGAGGAGGC-3′
Gapdh	F	5′-CAGATTCCAGGCCGAATGCG-3′
	R	5′-CTGGTTCACGAGACAGGTCC-3′

Supplementary Movies

Movie S1. Motility of wild-type spermatozoa.

Movie S2. Motility of *Slc22a14*$^{-/-}$ spermatozoa.
